# Identification and characterization of probiotic lactic acid bacteria isolated from traditional persian pickled vegetables

**DOI:** 10.3205/dgkh000300

**Published:** 2017-09-28

**Authors:** M.M. Soltan Dallal, S. Zamaniahari, A. Davoodabadi, M. Hosseini, Z. Rajabi

**Affiliations:** 1 Food Microbiology Research Center, Tehran University of Medical Sciences (TUMS), Tehran, Iran; 2 Division of Food Microbiology, Department of Pathobiology, School of Public Health, TUMS, Tehran, Iran; 3 Department of Microbiology, School of Medicine, Babol University of Medical Sciences, Babol, Iran; 4 Medical Statistics, Department of Epidemiology and Biostatics, School of Public Health, Tehran University of Medical Sciences. Tehran, Iran

**Keywords:** pickled vegetables, lactic acid bacteria, probiotic

## Abstract

**Background:** The pickle, a traditional fermented product, is popular among Iranians. Much research has been conducted worldwide on this food group. Due to a lack of related data in Iran, this study was conducted to isolate and identify dominant lactic acid bacteria (LAB) in pickles and salted pickles.

**Materials and methods:** Seventy samples were collected from different regions of Iran. The isolated bacteria were identified as LAB by Gram staining and catalase by using MRS agar. Then, those strains were identified at the species level by physiological tests (e.g., gas production from glucose, arginine hydrolysis, CO_2_ production from glucose in MRS broth, carbohydrate fermentation) and growth at temperatures of 15°C, 30°C, and 45°C in MRS broth for 3 days. The probiotic characteristics of these bacteria were studied using acid and bile tolerance. The corresponding results were verified using PCR analyses of the 16S rDNA region.

**Results:** 114 presumptive lactic acid bacteria (LAB) with Gram-positive and catalase-negative properties were obtained from the samples. The results revealed that all isolated bacteria were identfied as *Lactobacillus (L.) plantarum*,* L. brevis*, *L. pentosus*, *L. casei*, *L. paracasei *and* Leuconostoc mesenteroides*. The predominant LAB in these pickles was *L. plantarum*, which was isolated from most of the samples. Among the 114 LAB, 7 isolated species have probiotic potential. Six out of seven were recognized as *L. plantarum* and one remained unidentifiable by biochemical testing. PCR analysis and sequencing of the 16S rDNA region using 27f and 1522r primers showed that all of the probiotic strains were *L. plantarum*.

**Conclusion:** The results of this study showed that the dominant LAB in traditional Persian pickled vegetables are *L. plantarum*, *L. brevis*, *L. pentosus*, *L. casei*, *L. paracasei,* and *Leuconostoc mesenteroides*. Moreover, *L. plantarum* was recognized as a probiotic species in pickled vegetables. The raw data obtained from this study can be used in the pickling industry to improve the nutritional value of products.

## Zusammenfassung

**Hintergrund:** Das Essiggemüse, ein traditionelles vergorenes Produkt, ist bei der iranischen Bevölkerung sehr beliebt. Es wurden weltweit bereits viele Forschungsarbeiten zu diesem Lebensmittel durchgeführt. Bisher sind die Ergebnisse jedoch nur unzusammenhängend. Daher soll im Rahmen dieser Studie die Vielfalt der dominanten Milchsäurebakterien in Essig- und Salzgurken isoliert und identifiziert werden.

**Material und Methoden:** Es wurden 70 Proben aus verschiedenen Regionen des Irans analysiert. Die isolierten Bakterien wurden durch Gram-Färbung und Katalase-Testung sowie unter Verwendung von MRS-Agar als Milchsäurebakterien (LAB) identifiziert. Die Spezies dieser Stämme wurde durch physiologische Tests gesichert, wie Gasbildung von NH_3_ aus Arginin und von CO_2_ aus Glukose in Durham-Röhrchen mit MRS-Bouillon, das Wachstum bei Temperaturen von 15°C, 30°C und 45°C in MRS-Bouillon für 3 d sowie die Kohlenhydratfermentation. Die probiotischen Eigenschaften dieser Bakterien wurden unter Beobachtung ihrer Säure- und Gallentoleranz erfasst. Die Ergebnisse wurden mittels PCR-Analyse der 16S-rDNA Region verifiziert.

**Ergebnisse:** Durch experimentelle Untersuchungen wurden insgesamt 114 mutmaßliche LAB mit Gram-positiven und Katalase-negativen Eigenschaften identifiziert. Die Ergebnisse zeigten, dass alle isolierten Bakterien als *Lactobacillus (L). plantarum*, *L. brevis*, *L. pentosus*, *L. casei*, *L. paracasei* und *Leuconostoc mesenteroides* identifiziert wurden. In dem Gemüse wurde aus den meisten Proben überwiegend *L. plantarum* isoliert. Von den 114 LAB haben 7 isolierte Bakterien probiotisches Potenzial. Sechs von sieben wurden als *L. Plantarum* identifiziert, eine Spezies konnte nicht durch biochemische Untersuchungen detektiert werden. PCR-Analyse und Sequenzierung der 16S-rDNA-Region unter Verwendung von 27f- und 1522r-Primern zeigten, dass alle probiotischen Stämme *L. plantarum* zugeordnet werden können.

**Schlussfolgerung:** Die Ergebnisse zeigen, dass in traditionellem persischen Essiggemüse überwiegend die LAB *L. plantarum*, *L. brevis*, *L. pentosus*, *L. casei*, *L. paracasei* und *Leuconostoc mesenteroides* zu finden sind. Darüber hinaus wurde *L. plantarum* als probiotische Spezies in Essiggemüse detektiert. Die aus dieser Studie gewonnenen Rohdaten können in der Essiggemüse-Produktion verwendet werden, um den Nährwert der Produkte zu verbessern.

## Introduction

Lactic acid bacteria (LAB) is a large heterogenous group of Gram-positive and non-spore forming bacteria which was categorized based on a set of morphological, metabolic, and physiological characteristics [1]. The core of this microorganism group conventionally consists of *Streptococcus, Aerococcus, Pediococcus, Leuconostoc,* and *Lactobacillus* [2]. These bacteria are rarely grown on the culture media surface. Most of them grow in the carbohydrate-rich environments such as plants, fermented food, and mammalian mucosal surfaces such as the mouth, gut and vagina. LAB are a nutritionally fastidious bacteria, requiring rather complete nutritional supplementations. They chiefly need media enriched with vitamins, amino acids, and lipids for growth. 

This group of bacteria has been widely used in various industries, especially the food industry, due to certain specific characteristics [3]. Application of LAB as microbial culture in fermented meat production, dairy, and fermented vegetables is one of the oldest methods of processing food. Lactic acid fermentation is one of the most important processes in the food industry, because it increases shelf life and improves organoleptic properties. Successful fermentation depends on the quality of microbial growth, and as a result, LAB has been widely used. Due to the various antimicrobial substances produced by LAB, they can eliminate a wide range of undesirable organisms in the fermentation process. Also, in some fermented food products, LAB increases the vitamin content and the digestibility of raw materials [4].

The importance of lactic acid bacteria as a main group of probiotics is known to most scientists. Recently, numerous studies have been performed on the identification of probiotic microorganisms. Some of these studies were conducted for the production of probiotic dairy products, fruit juices, fermented agricultural products such as cabbage, squash, green beans, and olives. Although dairy products are the best known probiotic food source, many studies have shown that the fermented fruit and vegetable products can also be a good source of probiotics [5], [6]. According to Buckenhüskes et al. [7], plant-based fermented products will play an important role in the future due to:

- the high degree of hygiene and reliability in suppressing the growth of pathogenic bacteria,
- modern demands for natural and biological products,
- the fact that lactic acid bacteria and amino acids increase the nutritional value of raw products,
- improved taste and prevention of undesirable taste of glucosinolate,
- less energy required for preparation and storage,
- ease of processing raw material [7].

Based on the above, it seems that identification of lactic acid bacteria in pickled vegetables is necessary to use them to optimize industrial production.

## Materials and methods

### Sampling and measurement of pH 

Sampling was done from January 2013 to April 2014 from a total of 70 different types of traditional vegetable pickles: cabbage, garlic, eggplant, liteh, cabbage and carrots, olives and cucumber, with ten samples of each type. They were collected from different regions of Iran and transferred to the Food Microbiology Laboratory, Faculty of Health, Tehran University of Medical Sciences. Then, the pH of 10 ml of homogenised pickled vegetables was measured using a calibrated pH meter (Metrohm). 

### Culture preparation

To enrich media, 5 grams of samples were poured into flasks containing 45 ml MRS broth (Scharlau, Spain), then incubated at 35°C for 48 hours anaerobic. The samples were then subcultured on MRS agar (Scharlab; Barcelona Spain) and were incubated at 35°C for 48 hours anaerobic. The basic characteristics of bacterial colonies such as size, color and the margin were examined. After initial identification tests, Gram and catalase tests were performed. Plates containing catalase-negative and Gram-positive bacteria were selected and a single colony was transferred to MRS agar in order to multiply pure bacteria. The MRS agar was incubated at 35°C for 48 hours anaerobic [8], [9]. As the next step, the pure colonies were stored in MRS broth plus 18% glycerol at –20°C.

### Isolation and identification of LAB 

*L. plantarum* PTCC 1058 was used as positive control for performing biochemical tests. Next, in order to identify the species, the following tests were conducted:

- The ability to grow at various temperatures: Pure colonies grown in MRS agar were inoculated into tubes containing MRS broth and incubated in anaerobic jars at temperatures of 15°C, 30°C and 45°C for 72 hours [10]. Results were reported as positive if turbidity was observed after 72 h.
- Gas production from glucose: For this test, a modified MRS broth was prepared in which ammonium citrate was replaced by ammonium sulfate. Then it was poured into tubes containing Durham tube and sterilized. Subsequently, the pure colonies were grown on agar inoculated in this medium and incubated for 3 days in anaerobic jars at 35°C [10]. If CO_2_ was produced from the fermented glucose, the result was reported as positive.
- Carbohydrate fermentation: 13 types of sugar were used, as listed below (Table 1 (Tab. 1)). Using Jayne-William’s method, the sugar solutions were produced at concentrations of 20% and 10%, subsequently sterilized by filtration, and stored in refrigerator at 4°C as sugar stocks [11].
- Next, the MRS broth medium without glucose and with beef extract was prepared using chlorophenol red at a concentration of 0.004%. Its pH was adjusted to 8 and sterilized. A high-density bacterial suspension was made using saline, a sterile tube and pure colonies. Sugar stocks and sterile medium were mixed in the tubes to produce a final concentration of 0.5% glucose. After that, the prepared medium was mixed with bacterial suspension at defined density of about 10 λ in a microplate. Finally, to create anaerobic conditions, the microplate was sealed with sterile paraffin and was incubated at 30°C for 5 days. If the color changed from red to yellow, sugar fermentation was reported as positive [12], [13].
- Arginine hydrolysis test: For this test, a basal MRS broth medium without beef extract, but including 0.3% arginine and 0.2% ammonium citrate, was prepared. Then, the colonies grown in the basal medium were inoculated into the prepared medium and incubated in anaerobic jars at 35°C for 72 hours. This test was continued using Nessler's reagent (K_2_HgI_4_).

### Determination of probiotic potential

#### Biochemical method

In order to identify the probiotic potential of the isolated strains, two tests were conducted using *L. plantarum* PTCC 1058 as positive control:

- Acid tolerance was conducted according to Erkkila & Petaja’s method [14].
- Bile tolerance was performed using Gilliland’s method, so isolated LAB were cultured for 24 hours in MRS broth. 1% of the culture was added to MRS broth containing 0.3% bile acids (Oxgall). MRS without bile acids was used as the control. The optical density (OD) of theses cultures was determined before incubation at 620 nm using a spectrophotometer. Then, samples were incubated at 37°C for 8 hours. After that, optical density of the cultures was measured again at 620 nm [15].

#### Genotypic characterization method

Lastly, the results of the conventional methods (above) for identifying probiotic strains were confirmed by a molecular method. The identification of the selected isolates with acid and bile resistance was confirmed by 16S rDNA sequence analysis. Genomic DNA was extracted according to a previously described method [16]. The PCR primer sequences were as follows: forward primer, 5'-CTCGTTGCGGGACTTAA-3' and reverse primer, 5'-GCAGCAGTAGGGAATCTTC-3' (Bioneer, Korea) [17]. The reaction mixture consisted of 3 pmol of primers, 1.5 mM MgCl_2_, 0.2 mM dNTPs, 2 µl of genomic DNA, 5 µl 10X PCR buffer and 1.5 U of Taq DNA polymerase (Sinaclon, Iran) in a final volume of 50 µl. The PCR program started with an initial denaturation at 94°C for 2 min, followed by 30 cycles of 94°C for 30 s, 53°C for 1 min and 72°C for 1 min. PCR products were separated by Agarose gel electrophoresis (1.5% w/v) and visualized by staining with ethidium bromide. The PCR products of strains were sent to a sequencing company (Bioneer, Korea) and the 16S rDNA sequences were compared with known sequences in GeneBank using BLAST (http://www.ncbi.nlm.nih.gov/blast).

## Results

The main constituents of the sampled pickled vegetables are shown in Table 2 (Tab. 2).

In the first stage of enrichment in MRS broth, only 48 sample solutions (68.6%) showed turbidity. These samples were isolated to continue the test by culturing on MRS agar.

The frequency of stable LAB in pickled cabbage and carrot was 50%, in pickled olives, garlic, eggplant, and liteh it was 70%, in pickled cabbage it was 60%, and in pickled cucumber it was 90%. The average pH of these pickled vegetables was measured as 3.9.

A total of 114 LAB strains were isolated from 70 pickle samples. Biochemical tests were performed on the strains. The results were compared to the specifications mentioned in Bergey’s manual [12] and five groups of LAB strains were identified, *L. plantarum*, *L. brevis*, *L. pentosus*, *L. casei*, *L. mesenteroides,* and *L. paracasei*. About 17% of strains remained unidentified. About 65.5% of *L. plantarum* strains were able to grow at 15°C while just 34.5% of them were able to grow at both 15°C and 45°C . 

Thirty-two strains of the 114 isolated LAB were resistant to acid, so they were selected to undergo the bile resistance test. Finally, just 7 strains had both acid and bile tolerance characteristics.

PCR and sequencing verified the results of culture of culture method and showed *L. plantarum* and the others listed above, i.e. *L. brevis* and *L. pentosus* are probiotic.

## Discussion

Different kinds of pickled vegetables are one of the traditional popular fermented foods in the Middle East, especially in Iran. They are produced using traditional methods at small workshops and homes or by industrial methods in factories. Globally, different types of fermented fruit and vegetable products are made, such as miso, soya sauce, kimchi, tursu and a variety of other local pickled produce. Despite the benefits of these probiotic foods, due to the lack of standard production protocols, the pickle industry is not growing economically, in contrast to the industrial production of fermented dairy or meat products. Moreover, the pickle industry is highly dependent on local weather and harvest conditions [18]. Although it has been proven that the main microorganism responsible for the fermentation process in fermented vegetables is LAB, indigenous LAB in each region depend on the vegetable types, geographic location, temperature, conditions of harvest and method of preparation [19]. For example, in 2013, Tabatabaei et al. examined the isolation and identification of LAB in kimchi made in Iran and compared it to the kimchi made in South Korea [20]. They explained the difference between results as being dependent on the local vegetables and production conditions. Recently, because of the increasing importance of functional non-dairy products, many studies in different geographical areas are being performed to determine the identity of the indigenous LAB in these products. In the present study, the attempt was made to identify dominant LAB of seventy samples of traditional pickles using new methods of isolation, biochemical and molecular identification. 

There are several methods of identifying different probiotic LAB. In this study, the following tests were used: carbohydrate fermentation, gas production from glucose, hydrolysis of arginine, growth at different temperatures, and the resistance to acid and bile salts. 

In the case of carbohydrate fermentation, different carbohydrates may be used. For example, some authors used API kits [21], others tested without using a kit [22], [23], [24]. Fitzsimons used eight types of carbohydrate [25]. Ayhan et al. [23] assessed the fermentation of inulin, salicin, sorbitol, mannitol, and lactose. Pourahmad et al. [22] used eight types of carbohydrates. At some other researches, Scheirlinck and Vandamme in 2007 used 19 types of carbohydrate [24]. Jie Yu et al. [26] examined the fermentation of 19 types of carbohydrate, i.e., arabinose, cellobiose, esculin, galactose, gluconate, lactose, mannitol, mannose, melezitose, melibiose, raffinose, rhamnosus, ribose, salicin, sorbitol, surbose, sucrose, trehalose, and xylose. In the present study, 13 types of carbohydrates were used: arabinose, fructose, galactose, lactose, maltose, mannose, melibiose, raffinose, ribose, sucrose, cellobiose, sorbitol, and xylose. 

As mentioned above, 17% of the 114 isolates remained unknown. This could be due to mutations in one or more genes responsible for coding fermentation enzymes. It is possible that the carbohydrate needs of bacteria have changed over the years because of environmental or nutritional conditions [27]. In this study, six species, *L. plantarum*, *L. brevis*, *L. pentosus, L. casei*, *L. paracasei,* and *Leuconostoc mesenteroides,* were isolated. Most of these species were *L. plantarum*. Jie Yu et al. [26] examined 36 samples of vegetable pickles collected from six different regions of Sichuan Province, China. One hundred eighty-five strains were isolated: *L. plantarum* (81 strains), *L. pentosus* (38 strains), *L. brevis* (24 strains), *L. alimentarius* (16 strains), *L. paracasei* (9 strains),* L. sakei* (8 strains), *Pediococcus ethanolidurans* (5 strains), *Enterococcus thailandicus* (2 strains), *L. spicheri* (1 strain), and *Leuconostoc lactis* (1 strain). Those authors found *L. plantarum* to be the most stable species of LAB (43.8%) which is similar to the results of this study (50.9%) [26]. 

The most predominant LAB in kimchi (traditional pickle of a local cabbage, baechu) in South Korea were *Leuconostoc mesenteroides* and *L. plantarum* [28]. The present study also found this for *L. plantarum*, but not for *Leuconostoc mesenteroides*. This could be related to the different pH between the two products. Kimchi pH in the study by Choi et al. [29] was about 4.4 to 4.7, while the average pH of pickled vegetables in this study was about 3.96. A possible explanation may be the fact that *Leuconostoc mesenteroides* is less acid-resistant than *L. plantarum* [30]. 

In a study about fermented vegetables in India, Tamang et al. [31] confirmed by RAPD-PCR and phenotypic methods that sustainable LAB of these products include *L. brevis*, *L. plantarum*, *Pediococcus pentosaceus*, *P. acidilactici,* and *Leuconostoc fallax*, which agrees with the present study in terms of *L. plantarum*. In a study to identify the different types of LAB in vegetable pickles by culture-independent methods, Park et al. [32] confirmed that the variety of LAB depends on complex processes and different types of fermentation in vegetable pickles, so the diversity of LAB pickled vegetables in different geographical areas is not surprising. Yan et al. [33] showed that *L. plantarum* (43.6%), *L. pentosus* (19.1%), *Leuconostoc mesenteroides* (11%), and *L. brevis* (7.3%) were locally sustainable species in Chinese pickles. Similarly,* L. plantarum* (9.5%) and *L. brevis* (13%) were the most common LAB in the present study. 

In the current study, all isolates of *L. plantarum* grew at 15°C, and 34.5% of them grew at 45°C, which disagrees with Bergey’s manual [12]. In some other studies, Bahrami et al.* L. plantarum* was able to grow at 45°C as well [34]. For example, Bahrami et al. [34] assessed the biochemical characteristics, acid production and probiotic properties of *L. plantarum*,* L. curvatus,* and *L. paralimentarius* in studies on traditional sourdough. All three species grew at 15°C, and *L. plantarum* could also grow at 45°C. 

Unlike other previous studies on fermented vegetables [35], [36] which isolated different *Leuconostoc* species, only *Leuconostoc mesenteroides* (4.4%) was isolated in our study, which may be related to the different stages of fermentation. Studies conducted by Plengvidhya et al. [37] and Tao Xiong et al. [30] on sauerkraut showed that some species of LAB such as *Leuconostoc mesenteroides* and *Leuconostoc lactis* grow more rapidly at the beginning of the fermentation process and produce CO_2_ and lactic acid, which, accompanied by other activities of LAB, result in decreasing pH. Acid-resistant species, such as *L. plantarum* replace less acid-resistant species such as *Leuconostoc* spp. 

Compared to other studies in different countries which have isolated from one to eighteen different species [38], [39], LAB diversity in the present study seems low. Low diversity of species in pickled products can be caused by various factors, such as the kind of fruit and vegetables or vinegar used (traditional or industrial), method of preparation, the percentage of salt and vinegar, temperature, supply methods, geographic region, or the methods of the study. Chao et al. [39] showed that the diversity of LAB in fermented mustard depends on the harvest region. Preparation and maintenance temperature as well as salt content can change the variety of LAB in vegetable pickles [40].

Recent studies have shown that some LAB in fermented fruit and vegetable are resistant to acid and bile, similar to the LAB found in animal sources, so these products can be used as a suitable carrier for probiotics [41], [42]. For this reason, in the next phase of this study, probiotic tests were performed and 7 probiotic strains were isolated. In contrast, the biochemical tests isolated 6 strains of *L. plantarum* and one of the 6, was unclear. Thus, the probiotic species were assessed by molecular analysis to identify them more exactly. There are various methods of using molecular markers to more precisely identify lactobacilli, such as RAPD [25], [43], DNA-DNA hybridization as well as comparing the nucleotide sequence of 16S rDNA [21], [43], [44]. The two latter methods are more accurate than the others [43]. In this study, 16S rDNA sequencing was used and compared with the NCBI database to confirm the probiotic strains identified through biochemical tests. The results of PCR and sequencing showed that all isolated probiotic species were *L. plantarum*. Enan et al. [45] isolated 61 strains of probiotic bacteria by testing 100 samples of different pickled vegetables through biochemical and molecular tests. *L. plantarum* was isolated as the dominant probiotic species, which corroborates the results of this study. In a study of olive fermentation using PCR-RFLP, Esmaili et al. [46] isolated probiotic *L. plantarum*.

With the increasing use of harmful chemical additives in the food industry, it has become increasingly important to perform research on producing food additives with no adverse health consequences. LAB can be one of the options considered for this purpose, also bearing in mind their favorable effects on health and role as a preservative to increase the shelf life of food. In addition, probiotic LAB can be used to produce functional foods. 

## Notes

### Acknowledgements

This paper is part of a research project approved by the Food Microbiology Research Center, Tehran University of Medical Sciences and Health Services Contract No. 24051. 

### Competing interests

The authors declare that they have no competing interests.

## Figures and Tables

**Table 1:**
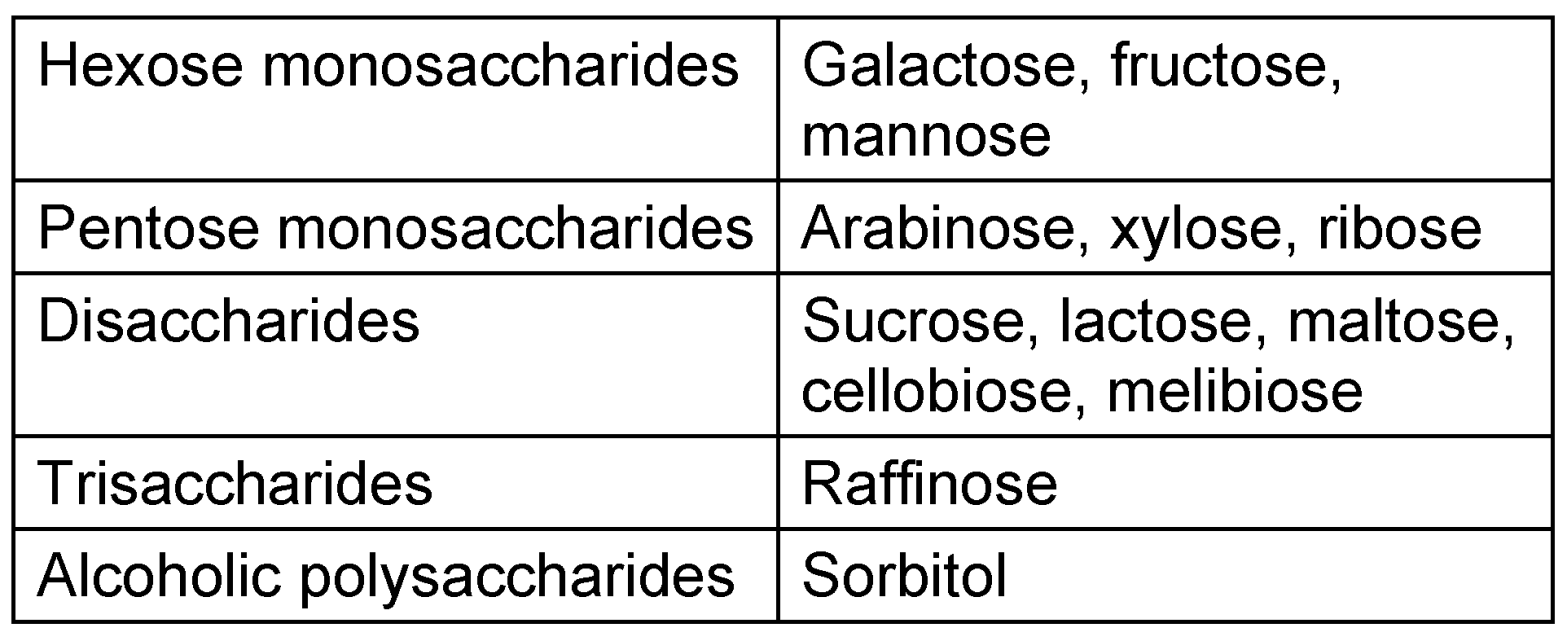
List of sugars used in the test

**Table 2:**
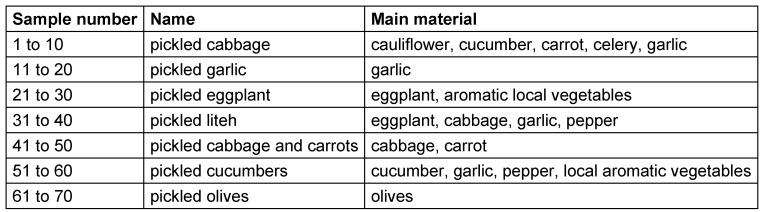
Main constituents of vegetable pickles
